# Plasma-Derived Hemopexin as a Candidate Therapeutic Agent for Acute Vaso-Occlusion in Sickle Cell Disease: Preclinical Evidence

**DOI:** 10.3390/jcm11030630

**Published:** 2022-01-26

**Authors:** Thomas Gentinetta, John D. Belcher, Valérie Brügger-Verdon, Jacqueline Adam, Tanja Ruthsatz, Joseph Bain, Daniel Schu, Lisa Ventrici, Monika Edler, Hadi Lioe, Kalpeshkumar Patel, Chunsheng Chen, Julia Nguyen, Fuad Abdulla, Ping Zhang, Andreas Wassmer, Meena Jain, Marcel Mischnik, Matthias Pelzing, Kirstee Martin, Roslyn Davis, Svetlana Didichenko, Alexander Schaub, Nathan Brinkman, Eva Herzog, Adrian Zürcher, Gregory M. Vercellotti, Gregory J. Kato, Gerald Höbarth

**Affiliations:** 1 Research and Development, CSL Behring AG, 3014 Bern, Switzerland; Thomas.Gentinetta@cslbehring.com (T.G.); Valerie.Verdon@cslbehring.com (V.B.-V.); Jacqueline.Adam@cslbehring.com (J.A.); lisa@ventrici.ch (L.V.); Monika.Edler@cslbehring.com (M.E.); Andreas.Wassmer@cslbehring.com (A.W.); Svetlana.Diditchenko@cslbehring.com (S.D.); Alexander.Schaub@cslbehring.com (A.S.); Adrian.Zuercher@cslbehring.com (A.Z.); 2 Division of Hematology, Oncology and Transplantation, Vascular Biology Center, Department of Medicine, University of Minnesota, Minneapolis, MN 55455, USA; belcher@umn.edu (J.D.B.); chenx028@umn.edu (C.C.); nguye088@umn.edu (J.N.); abdul023@umn.edu (F.A.); zhang074@umn.edu (P.Z.); verce001@umn.edu (G.M.V.); 3 CSL Behring Innovation GmbH, 35041 Marburg, Germany; Tanja.Ruthsatz@cslbehring.com (T.R.); Joseph.Bain@cslbehring.com (J.B.); Daniel.Schu@cslbehring.com (D.S.); Marcel.Mischnik@cslbehring.com (M.M.); 4 Bio21 Institute, CSL Ltd., Parkville, VIC 3052, Australia; Hadi.Lioe@csl.com.au (H.L.); Kalpeshkumar.Patel@csl.com.au (K.P.); Matthias.Pelzing@csl.com.au (M.P.); Kirstee.Martin@csl.com.au (K.M.); Roslyn.Davis@csl.com.au (R.D.); 5 Research and Development, CSL Behring, King of Prussia, PA 19406, USA; Meena.Jain@cslbehring.com (M.J.); Eva.Herzog@cslbehring.com (E.H.); 6 Research and Development, CSL Behring Ltd., Kankakee, IL 60901, USA; Nathan.Brinkman@cslbehring.com; 7 CSL Behring GmbH, Walcherstraße 1A/Stiege 1, 1020 Vienna, Austria; Gerald.Hoebarth@cslbehring.com

**Keywords:** hemopexin, hemoglobin, heme, sickle cell anaemia, vaso-occlusive crisis

## Abstract

People living with sickle cell disease (SCD) face intermittent acute pain episodes due to vaso-occlusion primarily treated palliatively with opioids. Hemolysis of sickle erythrocytes promotes release of heme, which activates inflammatory cell adhesion proteins on endothelial cells and circulating cells, promoting vaso-occlusion. In this study, plasma-derived hemopexin inhibited heme-mediated cellular externalization of P-selectin and von Willebrand factor, and expression of IL-8, VCAM-1, and heme oxygenase-1 in cultured endothelial cells in a dose-responsive manner. In the Townes SCD mouse model, intravenous injection of free hemoglobin induced vascular stasis (vaso-occlusion) in nearly 40% of subcutaneous blood vessels visualized in a dorsal skin-fold chamber. Hemopexin administered intravenously prevented or relieved stasis in a dose-dependent manner. Hemopexin showed parallel activity in relieving vascular stasis induced by hypoxia-reoxygenation. Repeated IV administration of hemopexin was well tolerated in rats and non-human primates with no adverse findings that could be attributed to human hemopexin. Hemopexin had a half-life in wild-type mice, rats, and non-human primates of 80–102 h, whereas a reduced half-life of hemopexin in Townes SCD mice was observed due to ongoing hemolysis. These data have led to a Phase 1 clinical trial of hemopexin in adults with SCD, which is currently ongoing.

## 1. Introduction

Sickle cell disease (SCD) is an autosomal recessive hemolytic anemia characterized by intermittent vaso-occlusion [1,2]. The key initiating event in the molecular pathogenesis of SCD is the polymerization of deoxygenated HbS, which leads to non-deformable, sickle-shaped red blood cells (RBCs). Expanding evidence over the last three decades indicate that the contribution of red cell rigidity is complemented by adhesiveness to endothelium and other circulating blood cells. The abnormal RBCs are more prone to lysis and endothelial adhesion because of activation of adhesion receptors (e.g., E-selectin, P-selectin, VCAM-1) and increased phosphatidylserine exposure and thus, increased interactions with leukocytes, platelets, and endothelial cells, resulting in the formation of heterocellular aggregates that initiate vaso-occlusion [3]. It is now known that the products of hemolysis promote acute and chronic vasculopathy through a combination of different mechanisms [4,5]. Extracellular hemoglobin in plasma can deplete nitric oxide signaling between endothelial cells and vascular smooth muscle cells, leading to acute and chronic vaso-constriction [6]. As this hemoglobin becomes oxidized or denatured, it is prone to release extracellular free heme [7,8]. Extracellular heme is a potent inducer of inflammation, known as a danger-associated molecular pattern (DAMP) molecule [9,10]. Heme promotes expression of pro-inflammatory cytokines and adhesion molecules by endothelium and blood cells. Prominently, heme induces exocytosis of Weibel-Palade bodies from endothelial cells, resulting in extensive endothelial cell surface exposure of P-selectin and von Willebrand factor. Both are known to play an important role in endothelial adhesiveness and vaso-occlusion [11]. Hemopexin, a high affinity heme scavenger protein in plasma, is significantly reduced in SCD patients compared to healthy controls [12,13,14]. In the sickle cell mouse, hemopexin reduces endothelial exposure of P-selectin and von Willebrand factor, stimulates hepatic heme oxygenase-1 (HO-1), which degrades heme to produce carbon monoxide, ferritin heavy chain, and biliverdin. The hemopexin and HO-1 pathways are associated with pronounced decreases in vascular inflammation and vaso-occlusion [15].

Vaso-occlusive crises (VOC) are the most common, painful complication of SCD and the main reason why patients seek medical care in hospitals. VOC may also be complicated by acute organ dysfunction, which manifests as acute chest syndrome, stroke or acute kidney injury, and these complications can be life-threatening. Current treatment options for acute VOCs are limited to supportive treatment, including hydration and analgesia, mainly with opioids [16]. Emergency department utilization and hospitalizations constitute a major economic impact of SCD, and the recurrent, unpredictable nature of the episodes diminishes quality of life [17,18]. Whilst therapies are available to reduce the frequency of VOC occurrence, such as the recently approved P-selectin inhibitor crizanlizumab [19], there remains an unmet need for a treatment to directly affect the biology of vaso-occlusion at the time of acute pain. Such a treatment might be expected to relieve pain, reduce health care utilization including hospital admission and improve overall quality of life.

We aimed to confirm the activity of hemopexin, through its effect on heme neutralization and removal, to alleviate endothelial cell inflammatory response and vaso-occlusion, and assess safety, tolerability, and pharmacokinetics in appropriate animal models to support clinical development.

## 2. Material and Methods

### 2.1. Plasma-Derived Hemopexin

Hemopexin was purified using the method as described in patent US 9534029B2. This method utilizes a modified Cohn Fraction IV-4 precipitate that is extracted, and impurities are precipitated using ammonium sulfate. Further purification was performed using hydrophobic interaction chromatography and nickel affinity chromatography. The final purified hemopexin was concentrated and formulated in phosphate buffered saline, pH 7.4.

### 2.2. Preparation of Heme-Albumin

The term “heme” is used generically to refer to both heme and hemin. These studies used hemin that was prepared immediately before use either dissolved only in NaOH or as heme-albumin. Briefly, 65 mg hemin (Frontier Scientific, Logan, UT, USA) was dissolved in 10 mL NaOH (0.1 M) at 37 °C, and then 15 mL of 20% human serum albumin (CSL Behring AG, Bern, Switzerland) was added. After 1 h of incubation at 37 °C, the pH of the solution was adjusted to pH 7.4–7.8 using 85% orthophosphoric acid. The 4 mmol/L heme-albumin solution was sterile-filtered (0.22 µm) and used immediately.

### 2.3. Preparation of Hemoglobin

Endotoxin-free ferrous human hemoglobin (Hb) was purified from pooled human plasma as described previously [20].

### 2.4. HUVEC Cell Culture Experiments

Human umbilical vein endothelial cells (HUVECs) were obtained from Promocell (Heidelberg, Germany). Cells were maintained in Endothelial Cell Basal Medium 2 (Promocell) including supplements ECBM2 (Promocell) under standard cell culture conditions of 37 °C, 5% CO_2_ and 95% humidity. Confluent cells were passaged using Accutase (Sigma-Aldrich Chemie GmbH, Buchs, Switzerland) into polystyrene cell culture plates as described below for stimulation assays. Confluent second-to-seven passage cells were used in all experiments.

### 2.5. HUVEC Stimulation and Flow Cytometry

Confluent HUVECs were harvested using Accutase (Sigma-Aldrich) according to the manufacturer’s protocol. Cells were transferred onto polypropylene 96-well cell culture plates (TPP Techno Plastic Products AG, Trasadingen, Switzerland) for stimulation and subsequent antibody labelling. Briefly, detached cells were preincubated with hemopexin (0, 25, 50, 100, 200, 400, 800 µmol/L) for 5 min before stimulation with heme (200 and 400 µmol/L) for 25 min at 37 °C. All stimuli were diluted in Endothelial Cell Basal Medium 2 (Promocell) incl. Supplement Pack ECBM2 (Promocell). Following stimulation cells were washed and labelled with APC anti-human CD62P (10 ug/mL, BioLegend, San Diego, CA, USA) in BD Pharmingen stain buffer (BD Biosciences, San Jose, CA, USA) for 25 min at 4 °C. APC Mouse IgG1, k isotype control antibody (10 ug/mL, BioLegend) served as antibody staining control. Only viable cells, as determined by 7-AAD (7-Aminoactinomycin; BioLegend) and AnnexinV (BioLegend) viability staining, were analyzed. HUVEC were analyzed on a FACSCanto II flow cytometer from BD Biosciences.

### 2.6. In Vitro Stimulation

HUVECs were preincubated with hemopexin as indicated for each experiment for 5 min before stimulation with hemin (50, 100 or 250 µmol/L) for 0, 8, 16 or 24 h, or as otherwise indicated. All stimuli were diluted in Endothelial Cell Basal Medium 2 (Promocell) incl. Supplement Pack ECBM2 (Promocell). Following stimulation, supernatants were collected and kept at −70 °C for Luminex assays (R&D systems, Minneapolis MN, USA) and cells were collected for Western blot analysis.

### 2.7. HO-1 Western Blot

Cells were washed twice in PBS-puffer pH 7.4 (Dr. G. Bichsel AG, Interlaken, Switzerland), lysed in RIPA buffer for 15 min on ice, and centrifuged to pellet debris. Cell lysates were run on SDS-PAGE and analyzed by Western blotting. Primary antibodies used were heme oxygenase 1 (ab 13243, rabbit, 1:1000, Abcam plc, Cambridge, United Kingdom) and beta actin (A2228, mouse, 1:10′000, Sigma-Aldrich). All secondary antibodies were purchased from Agilent Technologies AG (Basel, Switzerland): goat anti-mouse-HRP (horseradish peroxidase) and goat anti-rabbit-HRP. Data were acquired using Fusion Fx6 Imager (Witec AG, Heitersheim, Germany) and quantifications were done using Fusion FX6 Edge software (Witec AG).

### 2.8. Multiplex Cytokine Assays

A commercial human multiplex luminex kit (R&D Systems) was performed according to the manufacturer’s instructions. Briefly, 50 μL of sample (diluted 1:2) and standard were added in duplicates to the relevant wells and incubated with pre-mixed microbeads for 2 h on an orbital plate shaker at room temperature. The plates were washed three times with wash buffer and 50 μL of biotinylated detection antibody added per well. Samples were incubated for 1 h at room temperature on the plate shaker. Streptavidin–phycoerythrin (PE) solution (50 μL/well) was added, and plates incubated for a further 30 min at room temperature on a plate shaker, protected from direct light. Microparticles were resuspended by adding 100 µL of wash buffer. The assay was analyzed with a Bio-Plex 200 system (Bio-Rad, Hercules, CA, USA). The results were analyzed using Bio-Plex Data Pro software (Bio-Rad).

### 2.9. Animal Experiments

All animal experimental materials and methods are described in the accompanying Supplementary Material.

### 2.10. Statistical Evaluation

Analyses were performed with GraphPad Prism Software (version 9.0, San Diego, CA, USA). Comparisons of multiple treatment groups were made using one-way analysis of variance (ANOVA) (Dunnett’s multiple comparisons test).

## 3. Results

### 3.1. Hemopexin Protects against Endothelial Activation and Inflammation

**Figure 1:**
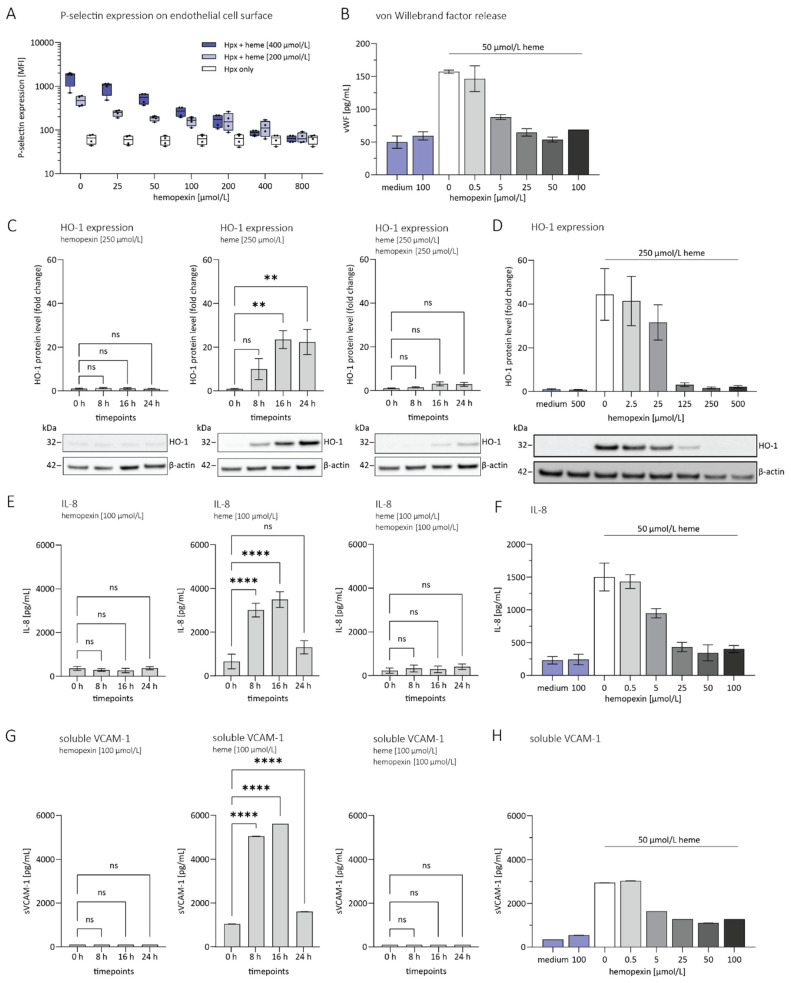
Hemopexin protects human endothelial cells from heme-driven activation and damage in vitro. (**A**) Cell surface P-selectin expression on human umbilical vein endothelial cells (HUVECs) upon stimulation with heme. HUVECs were incubated in different concentrations of hemopexin prior to stimulation with two concentrations of heme (400 and 200 µmol/L) for 25 min. Cell surface P-selectin expression was analyzed by flow cytometry. Data are presented as mean fluorescence intensity (MFI) visualized as Box and whiskers plots representing means ± min to max. Cumulative data of four independent experiments are shown. (**B**) Release of von Willebrand factor (vWF) upon heme stimulation (50 µmol/L) for 16 h in the presence of various concentrations of hemopexin (0–100 µmol/L). Data are expressed as the mean from three replicates ± SD. Blue bars: medium and hemopexin control, grey: heme stimulated HUVECs in presence of increasing concentration of hemopexin. (**C**) The plots represent HO-1 protein expression upon stimulation in presence of hemopexin, heme or the combination of hemopexin and heme assessed at different time points (0, 8, 16 and 24 h). Data are expressed as mean intensity ± SD from three independent experiments and a representative Western Blot figure is provided below each data plot. Concentrations are indicated for each plot. (**D**) Dose-dependent prevention of HO-1 expression at different concentrations of hemopexin (0–500 µmol/L) at a constant concentration of heme (250 µmol/L) after 16 h of stimulation was investigated. Data are expressed as mean intensity ± SD from three independent experiments and a representative Western Blot figure is provided below. (**E**,**G**) Heme-stimulated HUVECs released IL-8 and soluble VCAM-1 in a time-dependent manner. Time points investigated were 0, 8, 16 and 24 h and data are represented as mean ± SD from three replicates. (**F**,**H**) The release of IL-8 and soluble VCAM-1 after 16 h upon heme stimulation (50 µmol/L) in the presence of various concentrations of hemopexin was investigated (0–100 µmol/L). Data are expressed as the mean from three replicates ± SD. Blue bars: medium and hemopexin control, grey: heme stimulated HUVECs in the presence of increasing concentration of hemopexin. ** *p* < 0.01, **** *p* < 0.0001 versus control (cell culture medium). ns = not significant.

Heme alone strongly activates human umbilical vein endothelial cells (HUVECs). Heme-hemopexin complexes, however, do not show stimulatory potential. To test whether Weibel-Palade body degranulation can be assessed upon heme stimulation, we first investigated if cell surface P-selectin expression can be used as a readout in the presence of heme and various concentrations of hemopexin. HUVECs were preincubated with various hemopexin concentrations for 5 min followed by 25 min stimulation with heme. As shown in Figure 1A, increasing hemopexin concentrations during heme-mediated stimulation led to a dose-dependent reduction in cell surface P-selectin expression on HUVECs. Once an equimolar ratio between heme and hemopexin was reached, P-selectin expression was nearly abolished.

We found that heme promotes robust secretion of von Willebrand Factor (vWF) (Figure 1B) in cultured endothelial cells, which is, like P-selectin, stored within Weibel-Palade Bodies. Increasing hemopexin concentrations during heme mediated stimulation led to a dose-dependent reduction of vWF over 16 h. Like P-selectin, vWF secretion was mostly suppressed once an equimolar ratio between heme and hemopexin was reached.

We investigated the potential of hemopexin to prevent oxidative stress reflected by heme oxygenase 1 (HO-1) protein levels upon stimulation with heme in the presence or absence of hemopexin. As shown in Figure 1C, HUVECs were stimulated and harvested at different time points (0 h, 8 h, 16 h and 24 h). Cell extracts were analyzed by Western blot with a specific antibody against HO-1. HO-1 was significantly increased eight hours after stimulation with heme. In contrast, hemopexin reduced heme-induced HO-1 protein level, showing a protective role of hemopexin for endothelial cells mediated by heme scavenging.

Moreover, hemopexin suppressed release of two heme-inducible endothelial markers, IL-8 and soluble VCAM-1. IL-8 (Figure 1E) and soluble VCAM-1 (Figure 1G) were secreted upon incubation with heme for 8 h, but not by hemopexin alone. When hemopexin was added to the HUVECs 5 min before the stimulation with heme, IL-8 (Figure 1E) and soluble VCAM-1 (Figure 1G) were not secreted, and overall expression was comparable to control cells. In addition, hemopexin decreased expression of IL-8 and soluble VCAM-1 in a dose-dependent manner on heme-treated HUVECs (Figure 1F,H).

### 3.2. Duration of Protective Effect of Hemopexin Administered to Townes SS Mice at Different Doses and Different Times before VOC Challenge with Hemoglobin

**Figure 2:**
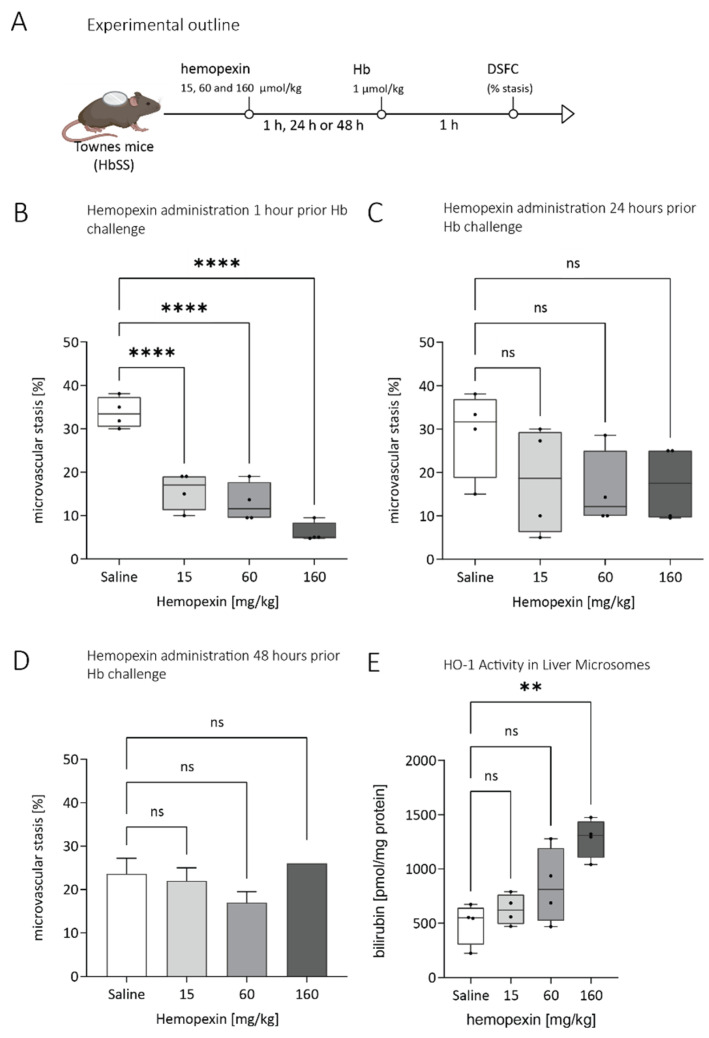
Dose range and durability of hemopexin effect administered at different times before hemoglobin triggered VOC. Dorsal skin-fold chambers were implanted onto Townes HbSS mice, and 20–24 flowing venules were selected in each mouse at baseline (time 0). (**A**) Prophylactic experimental setup: HbSS mice were infused with hemopexin (15, 60 and 60 mg/kg) or saline vehicle at baseline after selection of flowing venules. Hemoglobin (Hb, 1 μmol/kg) was infused 1, 24, or 48 h after hemopexin administration and microvascular stasis (% non-flowing venules) was measured in the same venules 1 h after Hb infusion. (**B**) Stasis data where hemopexin was administered 1h before Hb challenge (*n* = 4/group), (**C**) 24 h before Hb challenge (*n* = 4/group) and (**D**) 48 h (*n* = 2/group) before Hb challenge (**E**) Hemopexin treatment indicates a trend to increased HO-1 activity. Livers from HbSS mice treated with hemopexin and challenged after 1 h with Hb (1 μmol/kg) were removed and flash frozen 4 h after Hb infusion. Hepatic microsomes were used to assess HO-1 activity via bilirubin production. Box and whiskers plots represent means ± Min to Max. ** *p* < 0.01, **** *p* < 0.0001 versus HbSS (saline treated). ns = not significant.

We determined the duration of the effect of a single dose of hemopexin on microvascular stasis in Townes HbSS SCD mice over three different time intervals. Hemopexin was injected at three different doses, each at three time points before the mice were challenged with a single dose Hb injection (1 µmol/kg). We assessed stasis at 1 h after the Hb injection. Hemopexin prevented stasis in a dose-dependent manner when administered 1 h prior to the Hb injection, but not when given 24 or 48 h prior to Hb injection (Figure 2).

We measured HO-1 activity in liver microsomes as a measure of heme internalization and response. For this purpose, the livers from the microvascular stasis experiment described above were harvested after 4 h post-Hb treatment and microsomal isolation was performed. Confirming and extending previous studies [15], increased HO-1 activity was observed at the highest dose (160 mg/kg), demonstrating that hemopexin acts as a carrier to deliver additional heme into the liver for degradation (Figure 2D).

### 3.3. Protective Effect of Hemopexin Administration at Different Doses after VOC Challenge with Hemoglobin or Hypoxia

**Figure 3:**
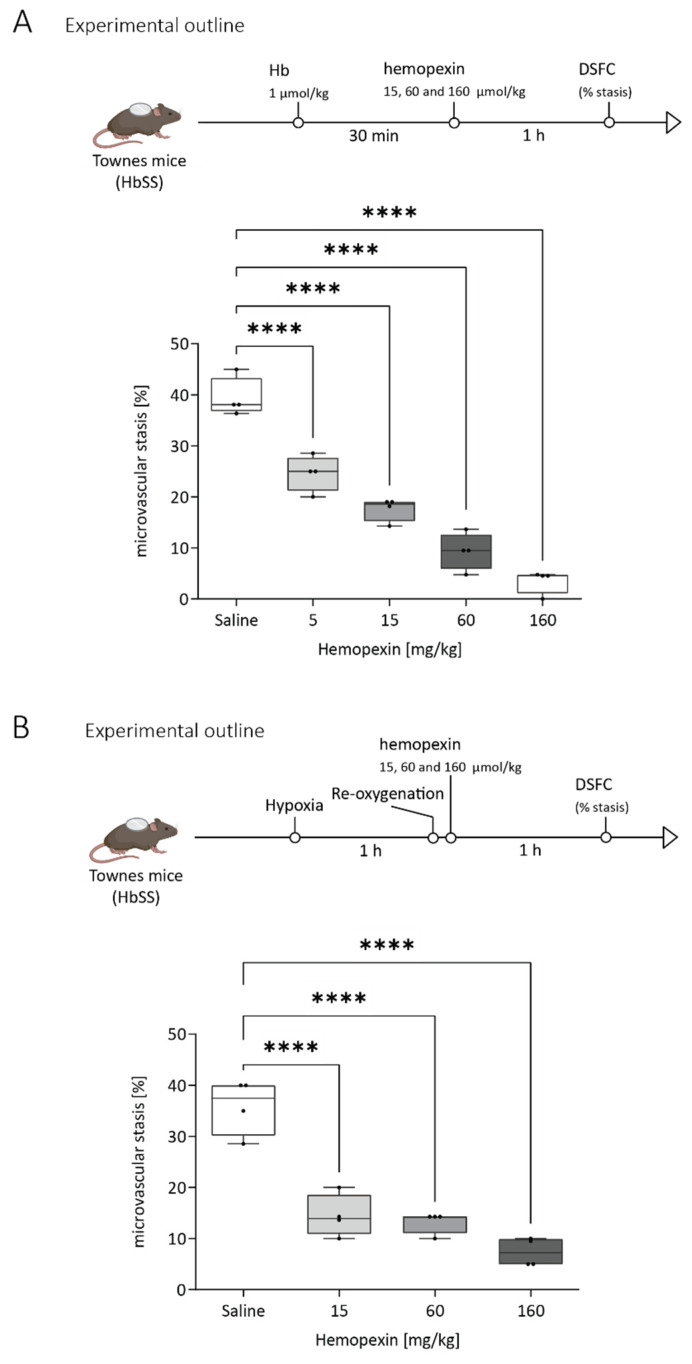
Hemopexin dose-dependent decrease in stasis after hemoglobin or hypoxic challenge. (**A**) Therapeutical experimental setup: Dorsal skin-fold chambers were implanted onto Townes HbSS mice and 20–24 flowing venules were selected in each HbSS mouse at baseline (time 0). Microvascular stasis was induced by hemoglobin (1 µmol/kg) infusion. Thirty minutes after the Hb challenge, animals were infused with hemopexin (5, 15, 60 or 160 mg/kg) or saline vehicle. Microvascular stasis (% non-flowing venules) was measured in the same venules 1 h after hemopexin infusion (*n* = 4/dose) (**B**) Therapeutical experimental setup: similar to (**A**) except that microvascular stasis was induced by hypoxia-reoxygenation (H/R, 7% O_2_ /93% N_2_) for 1 h followed by room air for 5 min with subsequent infusion of hemopexin (15, 60 or 160 mg/kg) or saline. Microvascular stasis (% non-flowing venules) was measured in the same venules 1 h after hemopexin infusion (*n* = 4/dose). Box and whiskers plots represent means ± Min to Max. **** *p* < 0.0001 versus SS (saline treated). ns = not significant.

We tested whether hemopexin could prevent stasis after the stasis was triggered, simulating a potential treatment approach in SCD patients with vaso-occlusive crises. Similar to above, Townes HbSS mice were challenged with hemoglobin (1 µmol/kg) and after 30 min, they were infused with hemopexin at the indicated doses, and compared to a saline negative control. Hemopexin provided a clear reduction of stasis in a dose-dependent manner 1 h after hemopexin infusion (Figure 3A).

Next, we tested whether the protective effect of hemopexin could extend to stasis triggered by an alternative stimulus. SCD mice were challenged with hypoxia-reoxygenation (H/R, 7% O_2_ /93% N_2_) for 1 h followed by room air and treated with saline or hemopexin at the indicated doses 5 min after return to room air. Hemopexin again reduced stasis in a dose-dependent manner, measured 1 h after hemopexin infusion (Figure 3B).

### 3.4. Pre-Complexed Hemopexin with Heme Does Resolve Hemoglobin Induced Stasis in a Dose-Dependent Manner

**Figure 4:**
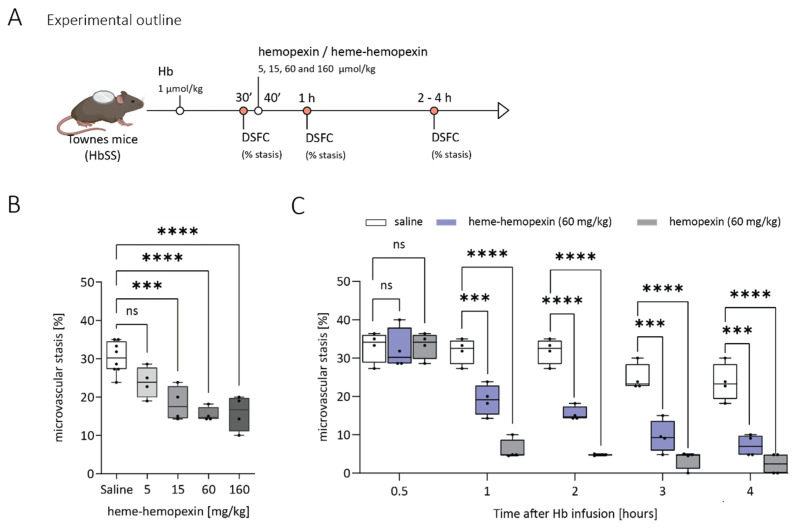
Hemopexin complexed to heme shows a dose-dependent reduction in stasis, but to a lower degree than apo-hemopexin. (**A**) Experimental treatment setup. Dorsal skin-fold chambers (DSFCs) were implanted onto Townes HbSS mice and 20–24 flowing venules were selected in each HbSS mouse at baseline. Microvascular stasis was induced by hemoglobin (Hb, 1 µmol/kg) and measured in the same venules 30 min after Hb infusion. At 40 min after the hemoglobin challenge, animals were infused with hemopexin, equimolar heme-hemopexin, or saline vehicle. (**B**) Microvascular stasis after HbSS mice (*n* = 4 mice/dose) were challenged with Hb and treated with equimolar heme-hemopexin complexes (5, 15, 60, 160 mg/kg hemopexin) or saline was assessed. Data shown from the 2 h time point (after Hb challenge) are presented as Box and whiskers plots representing means ± min to max. (**C**) Microvascular stasis readouts after HbSS mice (*n* = 4/dose) were challenged with Hb and treated with hemopexin (60 mg/kg) or heme-hemopexin (60 mg/kg) 40 min after Hb. Stasis was measured at 30 min after Hb (pre-hemopexin), and 1, 2, 3, and 4 h after Hb (post-hemopexin). Data are presented as Box and whiskers plots representing means ± min to max. White boxes: saline; blue boxes: equimolar heme-hemopexin (60 mg/kg); dark grey boxes: hemopexin (60 mg/kg). *** *p* < 0.001 and **** *p* < 0.0001 versus SS (saline treated).

We evaluated whether the protective effect of hemopexin on microvascular stasis would be prevented by pre-complexing hemopexin to heme, impeding its expected scavenging activity after injection. We infused different doses of equimolar heme-hemopexin (5, 15, 60 and 160 mg/kg) or saline 40 min after the intravenous hemoglobin challenge (1 µmol/kg). As expected, injection of saline control did not affect stasis (Figure 4B). Surprisingly, there was a clear dose-dependent effect of heme-hemopexin, similar to apo-hemopexin.

We directly compared the protective effect of a single dose (60 mg/kg) of apo-hemopexin versus an equimolar heme-hemopexin (60 mg/kg) complex over the different time points (Figure 4C). Heme-hemopexin was protective against stasis over a 1 to 4 h time interval, although less potently than apo-hemopexin. This unexpected finding suggests an inherent protective activity of hemopexin-heme complexes that is distinct from reducing levels of free heme.

### 3.5. Hemopexin Pharmacokinetics in Animal Models

**Figure 5:**
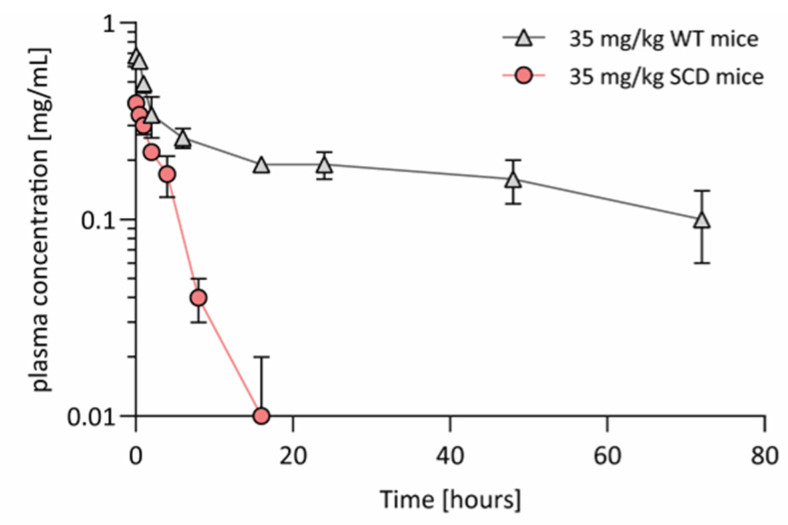
Differences in pharmacokinetics of hemopexin in SCD mice compared to WT mice. Hemopexin plasma concentrations in Townes HbSS and WT mice and rats upon intravenous administration of human hemopexin (*n* = 3/time point; 35 mg/kg). Individual data points represent mean concentration values ± SD.

We measured pharmacokinetics of hemopexin in WT versus hemolytic SCD mice. In comparison to C57BL/6 WT mice, Townes HbSS mice showed a markedly decreased C_max_ (0.41 mg/mL vs. 0.70 mg/mL) associated with a markedly increased clearance (23.0 vs. 1.6 mL/kg/h) and consequently decreased half-life (7 vs. 58 h) and mean residence time (MRT; 5 vs. 81 h) following IV administration of hemopexin (Figure 5 and Supplementary Table S1). Similarly, the area under the curve (AUC_0-inf_) was 14-fold higher in C57BL/6 WT- compared to Townes HbSS mice (21.8 vs. 1.52 h·mg/mL). PK parameters of hemopexin in rats were comparable to that in C57BL/6 WT mice-C_max_ (0.74 mg/mL), clearance (1.78 mL/kg/h), half-life (58 h) and AUC_0-inf_ (19.6 h·mg/mL) (Supplementary Figure S1B). The shorter half-life in Townes HbSS mice compared to the healthy rodent species (7 h vs. 58 h) suggests an increased binding of hemopexin to accessible heme in SCD, resulting in target-mediated disposition of hemopexin.

In cynomolgus monkeys, C_max_ and AUC of hemopexin increased slightly less than dose-proportionally over the dose range of 50–500 mg/kg (C_max_ from 1.6 to 10.4 mg/mL and AUC_0-inf_ from 84.4 to 587 h·mg/mL). A slightly higher total clearance (CL) (0.85 vs. 0.66 vs. 0.61 mL/h/kg) and a slightly higher volume of distribution at steady state (V_SS_) (105 (high dose) vs. 68 (mid dose) vs. 59 mL/kg (low dose)) was observed at the high dose compared to the intermediate and low dose. The terminal half-life following IV administration increased with dose from 80 to 103 h (Supplementary Table S1 and Supplementary Figure S1C).

After repeated IV administration to Townes HbSS mice, hemopexin plasma levels accumulated up to the third dose, reached a plateau and declined steadily over the following 20 h. The accumulation ratio was 2.63. The geometric means for AUC_0-inf_ (h·mg/mL) and AUC_0-last_ (h·mg/mL) were 345 and 268, respectively. MRT was 21.4 h, the half-life was 13.7 h, and the IVR was 93%. For heme-hemopexin complex, the highest plasma levels were measurable 5 min after the first dose of hemopexin. Levels declined slightly until they reached a steady state after the last dose up to the last time point measured. Total heme plasma concentrations first declined slightly and showed an increase towards the end of the concentration-time profile (Supplementary Table S1 and Supplementary Figure S2).

Following repeated dosing in Townes HbSS mice, increasing hemopexin concentration levels correlated with decreasing total heme plasma levels. The increase of total heme levels towards the end was probably due to the saturation of the clearance mechanism (i.e., complex internalization into the liver) and ongoing hemolysis. No clinically adverse effects were observed. Hematology, clinical chemistries, and urinalysis were unaffected by hemopexin administration.

### 3.6. Hemopexin Is Well-Tolerated in Animal Models

Exploratory tolerability studies showed that daily IV administrations of hemopexin for two weeks to C57BL/6 wild-type mice were well tolerated and produced no adverse clinical observations at 160 mg/kg. While there were no findings after the first dosing occasions, high dose IV animals (500 mg/kg) showed a short-lived, transient clinical reaction, i.e., reduced mobility and body temperature to the treatment on Day 8. There was no clear evidence of treatment-related changes to hematology or clinical chemistry parameters in any dose group at the end of the dosing period. In addition, daily IV administration of hemopexin for two weeks or six days (500 mg/kg only) to Townes HbSS mice was generally tolerated. Similar to the reaction observed in C57BL/6 wild-type mice, immunological reactions were observed in some dosing groups after repeat administration, which are considered to be a result of the application of the heterologous human protein.

Toxicity studies in rats and cynomolgus monkeys showed that repeated IV administrations of hemopexin were well tolerated at doses of 50, 150 and 500 mg/kg every other day for two weeks. The no observed adverse effect levels were set at the highest dose levels (500 mg/kg) in both species. Systemic exposure of hemopexin to rats and cynomolgus monkeys was roughly dose-proportional and increased with the duration of dosing. Non-adverse and transient effects that returned to baseline levels after the 7-day recovery period included an increase of the beta-globulin serum levels in cynomolgus monkeys, an increase of the alkaline phosphatase (ALP) serum levels, which did not clearly follow the dose-response principle in rats, and a small but statistically significant decrease from baseline activated partial thromboplastin time (aPTT). Repeated administration of human hemopexin to rats and cynomolgus monkeys was associated with the development of anti-drug antibodies (ADAs) in some animals. This observation is an expected immune reaction after repeated application of the heterologous human protein to animals, and is considered not to be predictive of the clinical situation.

Safety pharmacology evaluation did not show any deleterious effects of hemopexin on cardiovascular and respiratory functions in cynomolgus monkeys and rats at doses up to and including 500 mg/kg.

## 4. Discussion

Extracellular heme released during intravascular hemolysis appears to play an important role in promoting the abundant inflammation that peaks during episodes of acute pain in SCD [21]. Published experimental evidence confirms a cause-and-effect relationship between extracellular heme, hemopexin availability and inflammatory and vaso-occlusive consequences. These findings are consistent with circumstantial observations in patients with SCD. Our data reported in this manuscript confirms that heme mediates a significant signal of inflammation and endothelial adhesiveness. In our experiments, hemopexin neutralizes the pro-inflammatory and pro-adhesive activity of heme in cell culture.

The presented cell culture data emphasizes both the danger signal activity of heme on endothelial cells and the efficient heme-neutralizing activity of hemopexin. Hemopexin-bound heme molecules are rendered nonreactive and consequently, hemopexin potently prevents the robust pro-inflammatory effect of heme on endothelial cells. This effect is reflected by reversion of heme-induced expression of endothelial activation markers back to baseline. Hence, our study suggests a protective role of hemopexin for endothelial cells exposed to elevated levels of cell-free heme due to intravascular hemolysis, a phenomenon seen in Hb-triggered pathophysiological disorders such as SCD. Our new data are highly consistent with previous publications that demonstrate the ability of heme to promote P-selectin expression on the cell surface and the protective effect of hemopexin in cultured cells [11,22,23].

In our SCD mouse model, hemopexin in a dose-dependent manner ameliorated vaso-occlusion regardless of whether the vaso-occlusion was triggered by hemoglobin or by a heme-independent trigger, hypoxia-reoxygenation. Interestingly, this effect was partially replicated by hemopexin pre-complexed with heme, suggesting that the beneficial effect is not limited to clearance of circulating heme and prevention of TLR4 signaling. The partial effectiveness of the hemopexin-heme complex is consistent with our prior published data implicating CO generation from heme degradation by HO-1 as playing a role in the relief of vaso-occlusion by hemopexin [15]. Alternatively, heme-mediated activation of the oxidant-sensor Nrf2 transcription factor is known to activate various anti-inflammatory genes and inhibit some pro-inflammatory genes [24]. It has been proposed that hemopexin can be recycled after heme transport to hepatocytes, which could also allow the pre-complexed hemopexin to contribute additional cycles of heme scavenging and clearance [25]. More experiments are needed to clarify the activity of hemopexin in clearance-dependent and independent mechanisms of resolution of vaso-occlusion.

Hemopexin demonstrated a favorable safety profile in pharmacologically relevant animal species, and the pharmacokinetic results were consistent with published analyses of hemopexin.

For people living with SCD experiencing vaso-occlusive crises, current treatment options are generally supportive, with pain relief dominated by opioids, which in themselves have significant side effects and may lead to longer-term issues with hyperalgesia, tolerance and dependence [26]. Little progress has been made to date in developing effective treatments for the acute vaso-occlusive crisis itself that addresses the underlying disease process [27]. Hemopexin treatment has the potential to reverse the downstream pathophysiological effects of heme on the development of vaso-occlusion, with the effect of reducing the extent and duration of the pain, and potentially reducing healthcare utilization, such as hospital admission.

## 5. Conclusions

**Figure 6:**
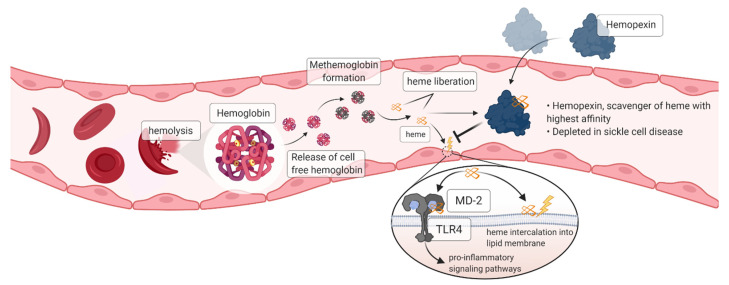
Current understanding of hemopexin Mode of Action. In patients suffering from sickle cell disease, repeated sickling cycles result in increased hemolysis. Hemolysis by-products such as heme leads to endothelial cell activation and oxidative stress, in part through the heme/TLR4/MD-2 signaling pathway and heme intercalation into cell membranes. Hemopexin, the natural scavenger of heme, is rapidly depleted under chronic hemolysis. Exogenous hemopexin supplementation proven to be effective in preventing endothelial cell activation in vitro and beneficial in the acute setting in vivo to reduce and resolve vaso-occlusion through its effect on heme neutralization and removal, induction of HO-1, and prevention of endothelial cell inflammatory responses (created with BioRender.com, accessed on 19 January 2022).

Based on the data presented above and the current understanding of the proposed mode of action (Figure 6), we conclude that hemopexin is a promising new candidate to treat acute vaso-occlusive crises in people living with SCD, and provides a basis for clinical development for this indication. A Phase 1 multicenter clinical trial to assess the safety, tolerability and pharmacokinetics of hemopexin (CSL889) in adults with SCD is currently ongoing (NCT04285827).

## Data Availability

Not applicable.

## Supplementary Materials

The following supporting information can be downloaded at: https://www.mdpi.com/article/10.3390/jcm11030630/s1, Figure S1: Rationale for treatment of acute VOC. PK parameter of hemopexin in SCD-, WT-mice, rats and monkeys. (**A**) Experimental outline and blood sampling time points. (**B**) Hemopexin plasma concentrations in SCD-, WT mice and rats upon intravenous administration of human hemopexin (N = 3/time point; 35 mg/kg) and (**C**) in cynomolgus monkeys following IV administration at three different dose levels (N = 3/dose; 50, 150 and 500 mg/kg). Individual data points represent mean concentration values ± SD.; Figure S2: Repeat dose injection in SCD mice. (**A**) Townes HbSS mice received 3 doses of 500 mg/kg hemopexin intravenously, each 4 h apart as indicated. Blood was sampled at baseline, 5 min before and 5 min after each hemopexin administration, and at 2, 4, 16, and 24 h after the last hemopexin administration. PK sampling used a Sparse Sampling approach, in which samples from different batches of mice were pulled together into 1 mean PK profile. N = 5 animals/time point. (**B**) The concentration-time profiles of hemopexin, hemopexin-heme complex, and total heme are illustrated. Hemopexin plasma levels accumulated up to the 3rd dose, reached a plateau for at least 4 h, and declined steadily over the following 20 h assessed. For heme-hemopexin complex, the highest plasma levels were measurable at 5 min after the first dose of hemopexin. Levels declined slightly up to 2 h after the last dose. Levels remained low until the last time point. Total heme plasma concentrations first declined slightly up to 2 h after the last dose of hemopexin administration and showed an increase towards the end of the concentration-time profile. Data is expressed as mean concentrations ± SD. Table S1: Summary of PK parameters after single dose IV administration of hemopexin (mouse, rat and cynomolgus monkey) and repeated dose administration.
