# Supplementary material for: Plasma-Derived Hemopexin as a Candidate Therapeutic Agent for Acute Vaso-Occlusion in Sickle Cell Disease: Preclinical Evidence
Source: J Clin Med. 2022 Jan 26;11(3):630. doi: 10.3390/jcm11030630 (PMC8836474; doi:10.3390/jcm11030630)
Supplement: Supplementary file 1 [file jcm-11-00630-s001.zip › jcm-1503400-supplementary.pdf]

DATA SUPPLEMENT

Plasma-Derived Hemopexin as a Candidate Therapeutic Agent for Acute Vaso-Occlusion in Sickle Cell Disease: Preclinical Evidence

Thomas Gentinetta[1,†], John D. Belcher[2,†], Valérie Brügger-Verdon[1], Jacqueline Adam[1], Tanja Ruthsatz[7], Joseph Bain[3], Daniel Schu[3], Lisa Ventrici[1], Monika Edler[1], Hadi Lioe[4], Kalpeshkumar Patel[4], Chunsheng Chen[2], Julia Nguyen[2], Fuad Abdulla[2], Ping Zhang[2], Andreas Wassmer[1], Meena Jain[5], Marcel Mischnik[3], Matthias Pelzing[4], Kirstee Martin[4], Roslyn Davis[4], Svetlana Didichenko[1], Alexander Schaub[1], Nathan Brinkman[6], Eva Herzog[5], Adrian Zürcher[1], Gregory M. Vercellotti[2], Gregory J. Kato[5,*] and Gerald Höbarth[7]

1 Research and Development, CSL Behring AG, 3014Bern, Switzerland.
2 Division of Hematology, Oncology and Transplantation, Vascular Biology Center, Department of Medicine, University of Minnesota, Minneapolis, MN 55455, USA.
3 CSL Behring Innovation GmbH, 35041 Marburg, Germany.
4 Bio21 Institute, CSL Ltd, Parkville VIC 3052, Australia.
5 Research and Development,CSL Behring, King of Prussia, PA 19406, USA.
6 Research and Development, CSL Behring Ltd., Kankakee, IL 60901, USA.
7 CSL Behring GmbH, 1020 Vienna, Austria.
* Correspondence: Gregory.Kato@cslbehring.com; Tel.: +1 (610) 290-7452; Fax: +1 (610) 290-0452
† These authors contributed equally to this work.

1. Supplementary Material and Methods

1.1. Animal Experiments

All animal experiments were approved by the respective Institutional Animal Care and Use Committees - i.e. the University of Minnesota, the participating contract research organization (CRO), and CSL. These studies utilized approximately equal numbers of male and female Townes-SS sickle mice on a 129/B6 mixed genetic background [1]. The Townes SS mice were created by knocking in human α and A$\gamma\beta$S globins into the deletion sites for murine α- and β-globins (Hbb$^{tm2(HBG1,HBB^{*})Tow)}$. SS-mice have anemia and an SS-RBC half-life of 2.5 days (d). Townes-AA control mice express normal human α and A$\gamma\beta$A globins with a 16 d RBC half-life [2]. All animals were housed in specific pathogen-free rooms to prevent infections on a 12 hour (h) light/dark cycle at 21°C. All animals were monitored daily including weekends and holidays for health problems, food and water levels and cage conditions. Littermates were randomly assigned to different treatment groups. No differences in endpoints were detected between male and female mice. All animals were included in each endpoint analysis and there were no unexpected adverse events that required modification of the protocol. Mice were aged 12-16 weeks and weighed 20-30 g.

Sprague-Dawley rats supplied by Charles River Laboratories Germany GmbH were used in all rat studies. An initial health check was performed upon delivery of the animals. Animals were acclimatized in the respective facility for a period of approximately 2 weeks before the start of dosing. Only animals without any visible signs of illness and who passed a health examination were used for the study. At study inclusion, all rats were >8 weeks old.

Captive-bred male and female cynomolgus monkeys were used for PK- and toxicity studies. The animals were kept in EU quarantine at the supplier's facilities until transfer to the CRO. Documentation (i.e. individual health certificates of the breeder with vaccination dates, tattoo number and tattoo number of the parents, certificate of health of the authorities at the release of the monkeys from EU quarantine, and other certificates) was

provided by the supplier. Upon arrival at the CRO, animals were subjected to a veterinary health check which included a detailed evaluation of each animal's condition. The animals were allowed to acclimatize for a period of 4 weeks before commencement of the study. At initiation of dosing, animals were 28-52 months old.

1.2. Induction of Vaso-occlusion and Pharmacological Treatment

Townes-SS mice were implanted with dorsal skin-fold chambers (DSFCs) as previously described [3]. On the same day or three days later, mice with DSFCs were anesthetized with a mixture of ketamine (106 mg/kg) and xylazine (7.2 mg/kg), placed on an intravital microscopy stage, and 20–24 flowing subcutaneous venules were selected and mapped. After baseline selection of flowing venules, mice were infused via the tail vein with hemoglobin (Hb) and hemopexin at the indicated doses and times. The same vessels were re-examined for stasis (no flow) at 1 h or as indicated and percent stasis (no flow) was calculated.

In other experiments, mice were challenged with hypoxia-reoxygenation (H/R, 7% O2 /93% N2) for 1h followed by room air for 5 min and treated with intravenous saline or hemopexin at indicated doses. Stasis was assessed at 1 hour after administration of hemopexin or saline.

1.3. Measurement of heme oxygenase enzyme activity in liver microsomes

Heme oxygenase (HO) activity was measured as previously described [4] in freshly isolated liver microsomes sonicated once for 10 seconds. Microsomes (2 mg) in 2 mM MgCl2, 0.1 M K2HPO4 buffer, pH 7.4 were added to the reaction mixture (400 µL, final) containing 2.5 µg of recombinant biliverdin reductase (Assay Designs), 2 mM glucose-6-phosphate, 0.2 U glucose-6-phosphate dehydrogenase, 50 µM hemin chloride (Frontier Scientific) and 0.8 mM NADPH (Calbiochem) for 1 h in the dark. Bilirubin that was formed was extracted into chloroform and measured by the delta OD at 464-530 nm (extinction coefficient, 40 mM-1 cm-1 for bilirubin). HO activity was expressed as pmol of bilirubin formed/mg microsomal protein/h.

1.4. Pharmacokinetic Assessment

To evaluate the pharmacokinetics (PK) of hemopexin, single-dose PK studies were performed following intravenous (IV) administration in Townes HbSS mice, wild type C57BL6 (WT) mice, Sprague Dawley rats at a dose of 35 mg/kg, and in cynomolgus monkeys at doses of 50, 150 and 500 mg/kg BW (Suppl. Fig. 1A). Furthermore, a repeat-dose PK study (up to 3 repeat doses each 4 hours apart at a dose of 500 mg/kg IV) in Townes HbSS mice was conducted. In addition to hemopexin, the concentration-time profiles of heme-hemopexin complex and total heme were described. Blood was sampled at baseline, 5 min before and 5 min after each hemopexin administration, and at 2, 4, 16, and 24 h after the last hemopexin administration. PK sampling used a Sparse Sampling approach, in which samples from different subgroups of mice were assembled together into 1 mean PK profile. Plasma was analyzed for hemopexin concentration, heme-hemopexin complexes and total heme. For hemopexin only, the following PK endpoints were estimated: $AUC_{0\text{-}inf}$ (ie, AUC from the last dose [8 h] to infinity) and $AUC_{0\text{-}last}$, mean residence time (MRT), initial volume of distribution (Vc), in vivo recovery (IVR), clearance (CL), C_{max} and terminal half-life ($t_{1/2}$). Plasma concentration-time profiles are shown in Suppl. Fig.1B and 2B. Townes HbSS mice were used because they express the human HbSS and mimic the major features of the human SCD (eg. sickled red blood cells (RBCs) with a half-life of 2.4 days vs normal RBCs with a half-life of 15.7 days in healthy mice). Quantification of total human hemopexin in mice, rats and cynomolgus monkeys was performed using LC-MS/MS.

1.5. Quantification of human hemopexin in mouse and rat plasma by LC-MS/MS

Ten microliters of plasma samples were placed into a clean Eppendorf tube followed by the addition of 75 µL MeOH to precipitate the protein. The methanol was removed after centrifugation and the pellet was air-dried and afterwards re-suspended in 50 mM NH4HCO3/0.16% ProteaseMAX containing heavy-isoptope labeled peptides, which are specific for human hemopexin and used as internal standards. Samples were incubated at 56°C/550 rpm for 45 min. Samples were reduced by adding 0.5 M DTT (56°C/550 rpm for 20 min) and then alkylated by adding 0.5 M IAA and incubated for 20 min at RT protected from light. Tryptic digestion was carried out at 37°C/550 rpm for 3 h and the samples were separated immediately on a C18 column (AdvanceBio Peptide Mapping, 2.1 x 250 mm). The measurements were conducted using an Agilent 6550 iFunnel QTOF mass spectrometer connected to an Agilent 1290 Infinity II HPLC instrument.

Data was analyzed by calculating the peak area of the analyte and the internal standard using Agilent MassHunter Quant software. A standard curve was created by Agilent MassHunter Quant where the average ratio of the analyte response to the internal standard response against concentration was plotted.

1.6. Quantification of human hemopexin in monkey serum by LC-MS/MS

Total human hemopexin concentration in cynomolgus monkey serum was quantified using liquid chromatography tandem mass spectrometry (LC-MS/MS) approach. Surrogate peptides that are specific to human hemopexin were used to achieve quantitation specificity, and isotopically labelled variants of the surrogate peptides were used as Internal Standard (IS) for assay normalization. Quantitation was achieved by comparison of the Multiple Reaction Monitoring (MRM) area ratio of the surrogate peptide to IS of the test samples against calibration standards, which were prepared by spiking known concentrations of human hemopexin to cynomolgus monkey serum.

All sample handling was performed on a plate. Briefly, 10 µL of serum samples were initially denatured with 1% sodium deoxycholate (DOC) (60 °C/ 800 rpm for 30 minutes), added with IS solution and then digested with Trypsin (37 °C/ 800 rpm for 2 hours). Digestion was stopped by adding 10% trifluoroacetic acid (TFA) solution and the mixture centrifuged for 10 minutes to remove DOC precipitate and 20 µL was injected into the LC-MS/MS for measurement. Column used: Waters XSelect Peptide CSH C18 XP, 2.1 x 100 mm; mobile phase A: 0.1% formic acid in water; mobile phase B: 0.1% formic acid in 98% acetonitrile/2% water; instrument: Agilent 1290 Infinity HPLC coupled to Sciex QTRAP 6500 triple quadrupole mass spectrometer; data acquisition software: Analyst 1.6.2; data analysis software: MultiQuant 2.1.

1.7. Total heme in mouse plasma

Total plasma heme concentration in mouse plasma was determined according to manufacturer's protocols using the QuantiChromTM Heme Assay Kit (BioAssay Systems). Briefly, 50 µL of sample (diluted in water 1:2) was placed into a 96-well plate. Assay reagent (200 µl per well) was added and incubated for 5 min at room temperature. Absorbance at λ400 nm was measured using a microplate reader (Synergy BioTek). Heme concentration was determined by comparison to a hemin standard curve (hemin preparation, seeMaterial and Methods).

1.8. Detection of heme-hemopexin complexes in mouse plasma

50 microliters of plasma sample were placed into a clean Eppendorf tube followed by the addition of 150 µL Buffer A (Multiple Affinity Removal Systems, Agilent). In a first chromatography step high abundant mouse proteins were depleted and carried out according to the manufacturer’s protocol on an Ultimate 3000SD HPLC attached to two LPG-3400SD quaternary pumps and a photodiode array detector (DAD) (ThermoFisher). Briefly, the diluted plasma sample was injected onto a multi affinity removal column depleting mouse albumin, IgG and transferrin (Mouse-3, 4.6 × 50 mm, Agilent) and separated with Buffer A (Multiple Affinity Removal Systems, Agilent) as the mobile phase at

a flow rate of 0.25 mL/min. Depleted plasma was collected into a fresh HPLC vial and re-injected and separated on a Diol-300 (3 μm, 300 X 8.0 mm) column (YMC CO Ltd.) with PBS, pH 7.4 (Bichsel) as the mobile phase at a flow rate of 1 mL/min. For all samples two wavelengths were recorded (λ = 280 nm and λ = 414 nm). The amount of heme-hemopexin complexes was determined by calculating the peak area of the complex (9 min retention time). Values from depleted plasma samples were interpolated by generating a standard curve based on peak area and plotted against the concentrations.

1.9. Toxicological Assessment

The potential toxicology of hemopexin was assessed in 2-week repeat dose tolerability studies in C57BL/6 wild-type and Townes HbSS mice and in 2-week repeat dose toxicity studies in Sprague Dawley rats and cynomolgus monkeys. Mice, rats and monkeys were selected as they represent standard animals for toxicological investigations with ample historical data. Mice were administered hemopexin intravenous (IV) at dose levels of 50, 160 and 500 mg/kg daily for 2 weeks. Sprague Dawley rats or cynomolgus monkeys received doses of 50, 150 or 500 mg/kg once every other day for a total of 14 days as a slow bolus IV injection, and followed by a 7 day recovery phase. Animals of the control group received 0.9% NaCl solution. Toxicological assessment included clinical signs, body weights, local tolerability, clinical pathology, ophthalmoscopy, toxicokinetics, development of anti-drug antibodies (ADAs), organ weights, and histopathological evaluation. In addition, safety pharmacological assessments of effects on the cardiovascular and central nervous system (CNS) systems were included in the repeat dose toxicology studies.

1.10. Pharmacokinetic evaluation

Pharmacokinetic analysis was conducted by either a non-compartmental approach (NCA), or two-compartmental modeling. In the latter case, the primary parameters (A, B, α and β), were computed by weighted least squares estimation, taking the measured standard deviations as weights. The terminal elimination rate λ either corresponds to the smaller constant of α and β was determined by linear regression through the terminal data points in the case of NCA. The maximum extrapolated plasma level was defined as C_{max}. The areas under the plasma concentration curve from zero to infinity ($AUC_{0\text{-}inf}$) and from zero to the last data point ($AUC_{0\text{-}last}$) were calculated either by numerically integrating the two-compartmental model, or by applying the trapezoidal method. Clearance was calculated as dose divided by $AUC_{0\text{-}inf}$, MRT as the area under the first moment curve from 0 to infinity ($AUMC_{0\text{-}inf}$) divided by $AUC_{0\text{-}inf}$ and terminal half-life as the natural logarithm of two divided by λ. Initial volume of distribution was calculated as dose divided by Cmax, steady state volume of distribution as clearance times MRT, and terminal volume of distribution as clearance divided by λ. IVR was defined as C_{max} divided by dose multiplied by plasma volume. For simplicity, plasma volume was assumed to be 40 mL/kg. Thus, an IVR of 100% corresponds to an initial volume of distribution of 40 mL/kg. Values below the lower limit of quantification (LLOQ), as well as clotted samples were excluded from analysis. The pharmacokinetic evaluation was conducted using WinNonlin (version 8.0, Pharsight Corporation, Mountain View, CA, USA), and Matlab (version R2019b, Mathworks, Natick, Massachusetts, USA).

2. Supplementary Results

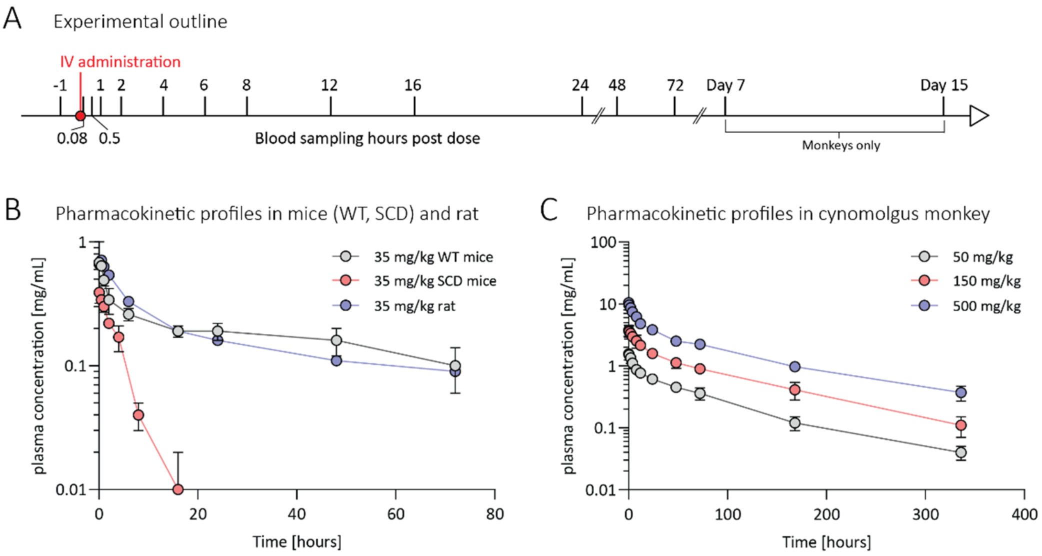

Supplementary Figure S1. Rationale for treatment of acute VOC. PK parameter of hemopexin in SCD-, WT-mice, rats and monkeys. (A) Experimental outline and blood sampling time points. (**B**) Hemopexin plasma concentrations in SCD-, WT mice and rats upon intravenous administration of human hemopexin (N = 3 / time point; 35 mg/kg) and **C**) in cynomolgus monkeys following IV administration at three different dose levels (N = 3 / dose; 50, 150 and 500 mg/kg). Individual data points represent mean concentration values ± SD.

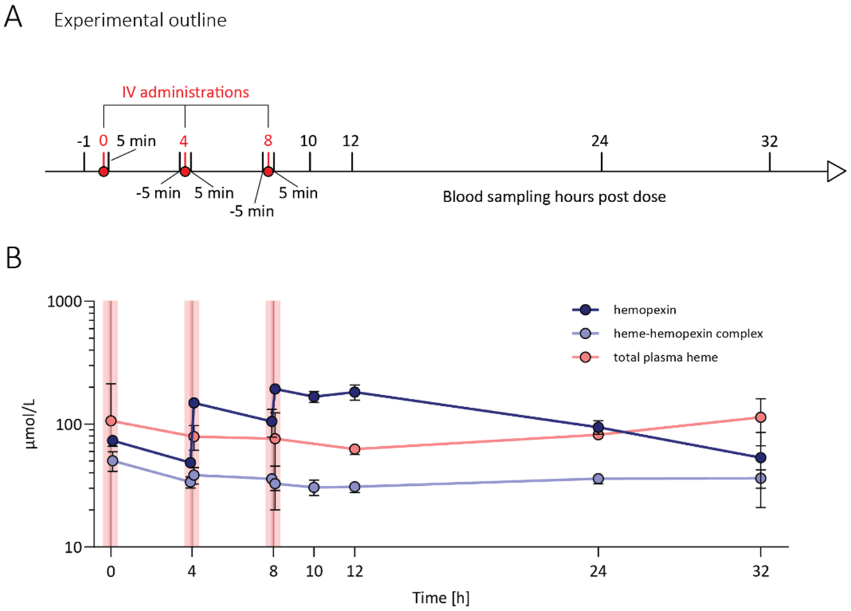

Supplementary Figure S2. Repeat dose injection in SCD mice. (A) Townes HbSS mice received 3 doses of 500 mg/kg hemopexin intravenously, each 4 hours apart as indicated. Blood was sampled at baseline, 5 min before and 5 min after each hemopexin administration, and at 2, 4, 16, and 24 h after the last hemopexin administration. PK sampling used a Sparse Sampling approach, in which samples from different batches of mice were pulled together into 1 mean PK profile. N = 5 animals / time point. (**B**) The concentration-time profiles of hemopexin, hemopexin-heme complex, and total heme are illustrated. Hemopexin plasma levels accumulated up to the 3rd dose, reached a plateau for at least 4 hours, and declined steadily over the following 20 hours assessed. For heme-hemopexin complex, the highest plasma levels were measurable at 5 min after the first dose of hemopexin. Levels declined slightly up to 2 h after the last dose. Levels remained low until the last time point. Total heme plasma concentrations first declined slightly up to 2 h after the last dose of hemopexin administration and showed an increase towards the end of the concentration-time profile. Data is expressed as mean concentrations ± SD.

Supplementary Table S1. Summary of PK parameters after single dose IV administration of hemopexin (mouse, rat and cynomolgus monkey) and repeated dose administration.

Species/strain	Mouse			Rat	Cynomolgus Monkey		
	Townes HbSS	C57BL/6 (WT)	Townes HbSS				
**Dose level (mg/kg)**	**35**		**500**	**35**	**50**	**150**	**500**
**$AUC_{0\text{-}inf}$ (h*mg/mL)**	1.52 (±0.2)	21.8 (±4.6)	345 (±38)	19.6 (±1.4)	84 (±17)	233 (±50)	587 (±47)
**C_{max} (mg/mL)**	0.41 (±0.0001)	0.7 (±0.03)	11.6 (±<0.1)	0.74 (±0.001)	1.6 (±0.316)	3.7 (±0.798)	10.4 (±0.960)
**Clearance (mL/kg/h)**	23.0 (±4.7)	1.6 (±0.3)	1.45 (±0.15)	1.78 (±0.12)	0.61 (±0.14)	0.66 (±0.12)	0.85 (±0.06)
**Half-Life term (h)**	7.23 (±2.6)	58 (±18)	13.7 (±3.5)	58.1 (±15.8)	79.9 (±5.8)	84.8 (±9.0)	102.5 (±15.1)
**MRT (h)**	4.82 (±1.3)	81 (±25)	21.4 (±4.4)	74.1 (±18.9)	n/a	n/a	n/a
**V_c (mL/kg)**	85.9 (±5.5)	47.3 (±2.2)	43.0 ±(<0.1)	47.1 (±3.9)	n/a	n/a	n/a
**V_{ss} (mL/kg)**	110.6 (±22.4)	130.5 (±15.4)	n/a	132.3 (±23.1)	58.7 (±7.7)	68.3 (±11.4)	104.7 (±9.4)
**V_z (mL/kg)**	239.6 (±88.2)	134.6 (±16.3)	n/a	149.5 (±28.8)	n/a	n/a	n/a
**IVR (%)**	46 (±0.001)	84.6 (±3.9)	93.0 (±<0.1)	85.0 (±0.1)	n/a	n/a	n/a

Values represent mean ± SD for each parameter. $AUC_{0\text{-}inf}$ = area under the plasma concentration time curve from time 0 extrapolated to infinity; CL = clearance; C_{max} = maximum plasma concentration; MRT = mean residence time; h = hour; IVR = in vivo recovery; N/A = not applicable; $t_{1/2}$ = terminal half-life; V_c = central volume of distribution; V_{ss} = volume of distribution at steady state; V_z = volume of distribution during the terminal phase.

References

1. Wu, L.-C.; Sun, C.-W.; Ryan, T.M.; Pawlik, K.M.; Ren, J.; Townes, T.M. Correction of Sickle Cell Disease by Homologous Recombination in Embryonic Stem Cells. *Blood* **2006**, *108*, 1183–1188.
2. Belcher, J.D.; Nath, K.A.; Vercellotti, G.M. Vasculotoxic and Proinflammatory Effects of Plasma Heme: Cell Signaling and Cytoprotective Responses. *ISRN Oxidative Med* **2013**, *2013*. https://doi.org/10.1155/2013/831596.
3. Kalambur, V.S.; Mahaseth, H.; Bischof, J.C.; Kielbik, M.C.; Welch, T.E.; Vilbäck, A.; Swanlund, D.J.; Hebbel, R.P.; Belcher, J.D.; Vercellotti, G.M. Microvascular Blood Flow and Stasis in Transgenic Sickle Mice: Utility of a Dorsal Skin Fold Chamber for Intravital Microscopy. *Am. J. Hematol.* **2004**, *77*, 117–125.
4. Balla, G.; Jacob, H.S.; Balla, J.; Rosenberg, M.; Nath, K.; Apple, F.; Eaton, J.W.; Vercellotti, G.M. Ferritin: A Cytoprotective Antioxidant Strategem of Endothelium. *J. Biol. Chem.* **1992**, *267*, 18148–18153.
